# AI-based prediction of pulmonary hypertension in COPD patients with cor pulmonale using clinical and CT features

**DOI:** 10.3389/fmed.2026.1869175

**Published:** 2026-09-01

**Authors:** Xiaohui Tan, Mian Luo, Rui Ma, Huanchi Liu, Lihua Xie, Rongchang Zhao

**Affiliations:** 1 The Third Xiangya Hospital of Central South University, Changsha, Hunan, China; 2 School of Computer Science and Engineering, Central South University, Changsha, Hunan, China

**Keywords:** artificial intelligence, chronic obstructive pulmonary disease, cor pulmonale, prediction mode, pulmonary arterial hypertension, volume of pulmonary arterioles

## Abstract

**Background and objectives:**

This study aimed to characterize COPD with clinically defined cor pulmonale and to develop clinical and contrast-enhanced CT-based AI-assisted models for its identification.

**Methods:**

We retrospectively enrolled 179 patients with COPD (90 COPD alone and 89 COPD with cor pulmonale). Clinical, laboratory, pulmonary function, electrocardiographic, and echocardiographic data were compared. To reduce incorporation bias, right ventricular, right atrial, and pulmonary artery measurements and electrocardiographic variables used in the operational case definition were excluded from candidate predictors. Variables associated with cor pulmonale in univariable analysis were entered simultaneously into multivariable logistic regression. A conservative sensitivity analysis additionally excluded mMRC score because dyspnea contributed to clinical case ascertainment. In 63 patients with diagnostic-quality CT angiography, pulmonary artery volumes were quantified using a U-Net model.

**Results:**

In the revised multivariable clinical model (*n* = 177), acute exacerbations in the past year (adjusted OR, 3.532; 95% CI, 1.893–6.593) and mMRC score (adjusted OR, 2.165; 95% CI, 1.363–3.439) were positively associated with cor pulmonale. Hypertension showed an inverse sample-specific association (adjusted OR, 0.333; 95% CI, 0.141–0.784). The revised model achieved an AUC of 0.888 (95% CI, 0.838–0.937), with 79.3% sensitivity and 87.8% specificity. The sensitivity model excluding mMRC score retained an AUC of 0.870 (95% CI, 0.816–0.923). The small pulmonary artery volumes Vd_12_, Vd_20_, Vs_15_, and Vs_30_ mm were lower in the cor pulmonale group as reported in the vascular analysis, and the AI-assisted vascular model yielded an AUC of 0.895 (95% CI, 0.820–0.970).

**Conclusion:**

A revised clinical model that excluded variables incorporated into the diagnostic definition retained good discrimination for clinically defined cor pulmonale. AI-assisted quantification of small pulmonary vessels may provide complementary non-invasive information.

## Introduction

Chronic obstructive pulmonary disease (COPD) is a chronic airway inflammatory disease characterized by abnormalities in the airways and alveoli, which can lead to persistent and progressive airflow limitation, causing a series of symptoms such as coughing, expectoration, and shortness of breath (1). As the disease progresses, COPD patients may develop chronic lesions in the lung parenchyma and pulmonary vessels, gradually increasing pulmonary circulation resistance, which can lead to right ventricular hypertrophy and dilation, and further develop into chronic cor pulmonale. Currently, chronic cor pulmonale is a major public health problem worldwide. The common pathophysiological process of chronic cor pulmonale is pulmonary hypertension (PH). Studies (2, 3) have shown that the incidence of pulmonary hypertension in COPD patients is closely related to the severity of the disease. Some COPD patients present with mild to moderate pulmonary hypertension, and the incidence of severe pulmonary hypertension is generally less than 10% (4). If pulmonary hypertension in COPD patients is not treated promptly and effectively, it may develop into cor pulmonale. Therefore, timely diagnosis of pulmonary hypertension in COPD patients is crucial to reduce the risk of concurrent cor pulmonale.

In the early stage of COPD with pulmonary hypertension, the clinical symptoms are often atypical. When patients show progressive dyspnea, fatigue, lower extremity edema, and palpitations, the disease has already progressed to severe cor pulmonale. Right heart catheterization (RHC) is the gold standard for the diagnosis and classification of pulmonary hypertension (5), but as an invasive procedure with potential complications, it has not been widely used in clinical practice (6). In addition, this examination requires high technical skills and is not suitable for early screening. Not all medical institutions have the conditions for RHC. Therefore, non-invasive diagnostic methods are necessary for the early detection of pulmonary hypertension in patients. Imaging is an indispensable part of evaluating disease characteristics and clinical diagnosis (7). Chest CT scan is a common examination method for COPD, which can not only show emphysema, bullae, and bronchial lesions, but also provide detailed descriptions of pulmonary hypertension and the pulmonary vascular features associated with it (8, 9). The typical CT features of pulmonary hypertension include sparse distal small vessels, increased proximal vessel volume, and increased arterial tortuosity (10). Pulmonary hypertension affects both large and small pulmonary vessels, so measuring small pulmonary vessels is also important for the diagnosis, severity assessment, and prognosis prediction of pulmonary hypertension (11, 12). Currently, most studies on pulmonary hypertension using CT images are based on the measurement of large vessels (13, 14), and the cross-sectional area of small pulmonary vessels as a two-dimensional measurement index may be affected by the cutting plane, so small pulmonary vessels are rarely used in the research of pulmonary hypertension.

In recent years, with the development of artificial intelligence (AI), deep learning technology has become increasingly mature, and its application in the field of medical image analysis has gradually increased. Because it can quantify the radiological features of different lung diseases, it is widely used in non-invasive diagnosis in imaging (15). Currently, there are few studies on the use of AI to assist in the diagnosis of pulmonary hypertension in medical images, and there is no research on the measurement of small pulmonary vessel volume by AI in the identification of pulmonary hypertension. This study first analyzes the differences in clinical characteristics between the simple COPD group and the COPD with cor pulmonale group to understand the clinical characteristics of COPD with cor pulmonale. And through the enhanced CT of the patient’s lungs, using a deep convolutional neural network, a three-dimensional pulmonary vascular network was reconstructed, incorporating parameters of small pulmonary vessels, to construct a model for identifying clinically defined cor pulmonale, with the aim of providing a potential non-invasive auxiliary approach for patients with COPD.

## Materials and methods

### Clinical data

The clinical data of COPD inpatients who admitted in the Third Xiangya Hospital of Central South University from 2017 to 2022 were retrospectively obtained. Inclusion criteria: meeting the COPD diagnostic criteria of the 2017 GOLD guidelines (16). For this retrospective study, clinically defined cor pulmonale was identified using operational criteria based on the available echocardiographic, electrocardiographic, and clinical records. These operational echocardiographic thresholds were selected with reference to published recommendations on right-heart echocardiographic assessment and pulmonary arterial hypertension evaluation (17, 18). Inclusion criteria for COPD with cor pulmonale: (1) having a clear diagnosis of COPD; (2) meeting one of the following echocardiographic diagnostic criteria for cor pulmonale: ① right ventricular dilation (RV) > 41 mm; ② pulmonary artery diameter (PA) ≥ 18 mm; ③ right atrial area > 18 cm^2^ or volume > 33 mL/m^2^; (3) meeting one of the following electrocardiographic diagnostic criteria for cor pulmonale: ① general right axis deviation, frontal plane mean electrical axis ≥ +90°; ② right bundle branch block (RBBB): prolonged QRS complex duration (≥120 ms), and presenting rsR’ morphology in lead V1; ③ increased P wave amplitude in leads II, III, and aVF (>2.5 mm), suggesting pulmonary P wave. (4) Having clinical manifestations of COPD and cor pulmonale: ① decreased exercise tolerance, fatigue after activity, exertional dyspnea, etc; ② physical examination: signs of cor pulmonale, such as lower extremity edema, jugular vein distension, and positive hepatojugular reflux sign, etc. Having clinical manifestations of COPD and cor pulmonale, and meeting (1) + (2), (1) + (3), or (1) + (2) + (3) can all diagnose COPD with cor pulmonale. Exclusion criteria: (1) having other pulmonary diseases, such as bronchiectasis, asthma, interstitial lung disease, etc; (2) having pulmonary hypertension caused by other factors, unstable or poorly controlled hypertension, heart disease; (3) having hematological diseases, severe liver and kidney diseases, tumors, etc; (4) incomplete data. This study received ethical approval from Third Xiangya Hospital of Central South University ethics committee (Approved by the Ethics Committee of The Third Xiangya Hospital, Central South University via expedited review (Approval No. 251072).

### Grouping

Based on the signs of chronic cor pulmonale indicated by the patients’ lung CT, electrocardiogram, echocardiogram, etc., the patients were determined to have or not have cor pulmonale, and were divided into the simple COPD group and the COPD with cor pulmonale group. At the same time, a normal healthy group was collected by matching age and gender.

### Data collection

Collect general patient information, the number of acute exacerbations in the past year, clinical symptoms, mMRC dyspnea score, and laboratory test data of the patients, including arterial blood gas, venous blood test indicators, pulmonary function, electrocardiogram, enhanced CT images of the lungs, and echocardiography results within 24 h after admission.

### Construction of an aI-assisted model for identifying cor pulmonale based on contrast-enhanced chest CT

#### Pulmonary artery vessel extraction

Step 1: Apply the OTSU method (19) and adaptive threshold algorithm to each cross-sectional image, followed by the removal of larger connected components to obtain smaller suspected vessel regions (Supplementary Figure 1).

Step 2: Remove noise from the initial suspected vessel regions based on manual annotation.

Step 3: Train the convolutional neural network (U-Net architecture) using the paired images and select the best-performing model on the test set to extract Pulmonary artery vessels from the overall data (Supplementary Figure 2).

The deep convolutional neural network for vascular segmentation selected in this study is shown in Supplementary Figure 2, with the main structure being the U-Net architecture. The U-Net used in this study implements the upsampling operation using the bilinear interpolation algorithm.

### Data processing and evaluation indicators of the model

The obtained paired data were randomly divided into train and test sets at a ratio of 8:2, with 800 and 200 samples in each set, respectively. The image preprocessing in this paper is. To divided into two parts: data augmentation and normalization objectively evaluate the test results of multiple models and data for pulmonary artery segmentation, this study selected six medical image segmentation evaluation indicators: accuracy (Accuracy), sensitivity (Sensitivity), specificity (Specificity), geometric mean (G-Mean), intersection over union (IOU), and Dice coefficient (Dice) (Supplementary Table 1 for details).

### Measurement of pulmonary vascular parameters

Pulmonary arterial hypertension is associated with reduced remodeling and dilation of small vessels, leading to increased pulmonary vascular resistance and pulmonary arterial pressure. According to the literature (20), the total volume of small vessels with pulmonary artery diameters of 12, 16, and 20 mm (denoted as Vd_12_, Vd_16_, and Vd_20_ mm, respectively) and the total volume of small arteries within 15, 30, and 45 mm from the pleural surface (denoted as Vs_15_, Vs_30_, and Vs_45_ mm, respectively) were calculated. The vascular tortuosity of the pulmonary artery, which is the ratio of vascular displacement to total length, was calculated and denoted as T%. The diameter of the main pulmonary artery (MPAD) and the diameter of the aorta (AOD) 1cm below the carina on the CT image were measured, and their ratio was calculated.

### Statistical methods

Statistical analyses were repeated using Python 3.13 with SciPy, statsmodels, and scikit-learn. Continuous variables were summarized as mean ± standard deviation or median (interquartile range), as appropriate, and compared using the independent-samples *t*-test or Mann-Whitney U test. Categorical variables were expressed as *n* (%) and compared using the chi-square test or Fisher exact test when appropriate. For the clinical logistic regression analysis, variables that directly contributed to the operational definition of cor pulmonale—right ventricular diameter, right atrial diameter, pulmonary artery diameter, right-axis deviation, right bundle branch block, and pulmonary P wave—were classified as diagnostic components and excluded a priori from candidate predictors. Univariable logistic regression was performed for the remaining clinical, laboratory, and pulmonary function variables. Variables with *P* < 0.05 were entered simultaneously into the multivariable model without automated stepwise selection. Variance inflation factors were calculated to assess multicollinearity. Because exertional dyspnea contributed to retrospective clinical case ascertainment, a conservative sensitivity analysis additionally excluded mMRC score. Complete-case analysis was used; two patients had missing values for acute exacerbations, leaving 177 patients in the multivariable analyses. Discrimination was assessed using receiver operating characteristic curves, area under the curve (AUC), and 95% confidence intervals estimated using DeLong’s method. Optimal thresholds were selected using the Youden index. Calibration was assessed using the Hosmer-Lemeshow test and Brier score. All tests were two-sided, and *P* < 0.05 was considered statistically significant.

## Results

### Comparison of clinical data between the two groups of patients

A total of 1,056 COPD inpatients admitted between 2017 and 2022 were screened; 877 were excluded, leaving 179 patients (90 with COPD alone and 89 with COPD and cor pulmonale). Recalculation from the source spreadsheet showed that hypertension was more frequent in the COPD-only group than in the cor pulmonale group (46.7% vs. 31.5%, *P* = 0.037), whereas diabetes did not differ between groups (12.2% vs. 10.1%, *P* = 0.654). Patients with cor pulmonale had more cough, expectoration, shortness of breath, fatigue, acute exacerbations, and higher mMRC scores. They also had higher PaCO_2_, hemoglobin, red blood cell count, and blood urea nitrogen, lower oxygen saturation, oxygenation index, and platelet count, larger right-sided cardiac and pulmonary artery measurements, and poorer pulmonary function (Table 1).

**TABLE 1:** Comparison of basic characteristics between the chronic obstructive pulmonary disease (COPD) group and the COPD with cor pulmonale group [M (Q1, Q3)/(±s)/*n* (%)].

Indicator	COPD group (*n* = 90)	COPD with cor pulmonale group (*n* = 89)	χ^2^/Z/*t*	*P*-value
Age (years)	70.96 ± 8.65	71.73 ± 8.84	−0.593	0.554
Height (cm)	165.00 (161.25, 169.50)	165.00 (163.00, 168.00)	−0.576	0.565
Weight (kg)	58.00 (55.00, 60.75)	59.00 (54.00, 62.00)	−1.099	0.272
Gender			1.039	0.308
Male	83 (92.2)	78 (87.6)	–	–
Female	7 (7.8)	11 (12.4)	–	–
Smoking	66 (73.3)	64 (71.9)	0.046	0.831
Drinking	32 (35.6)	28 (31.5)	0.402	0.526
Underlying diseases	88 (97.8)	87 (97.8)	0.000	0.991
Hypertension	42 (46.7)	28 (31.5)	4.345	0.037
Coronary heart disease	17 (18.9)	26 (29.2)	2.614	0.106
Diabetes	11 (12.2)	9 (10.1)	0.201	0.654
Chronic liver disease	8 (8.9)	4 (4.5)	1.382	0.240
Chronic kidney disease	3 (3.3)	3 (3.4)	0.000	0.989
Malnutrition	6 (6.7)	5 (5.6)	0.085	0.770
Clinical signs and symptoms				
Coughing	65 (72.2)	85 (95.5)	17.869	<0.001
Expectoration	64 (71.1)	82 (92.1)	13.153	<0.001
Shortness of breath	59 (65.6)	82 (92.1)	18.905	<0.001
Fever	9 (10.0)	6 (6.7)	0.619	0.431
Chest tightness	17 (18.9)	28 (31.5)	3.758	0.053
Fatigue	8 (8.9)	17 (19.1)	4.009	0.045
mMRC score (points)	2.00 (1.00, 2.00)	3.00 (2.00, 3.00)	−6.814	<0.001
Number of acute exacerbations in the past year	1.00 (0.00, 1.00)	1.00 (1.00, 2.00)	−6.871	<0.001
Laboratory test indicators				
pH	7.41 (7.37, 7.44)	7.40 (7.37, 7.43)	1.063	0.288
PaO_2_ (mmHg)	82.30 (67.38, 97.15)	80.10 (68.10, 88.40)	1.220	0.222
PaCO_2_ (mmHg)	38.30 (34.60, 46.38)	45.00 (39.00, 52.10)	−3.663	<0.001
SaO_2_ (%)	95.55 (93.85, 97.90)	94.10 (92.00, 96.80)	2.952	0.003
Oxygenation index (mmHg)	318.29 (251.81, 391.48)	282.76 (236.55, 335.52)	2.520	0.012
WBC (×10^9^/L)	7.05 (5.70, 9.37)	6.81 (5.12, 8.55)	1.395	0.163
Hb (g/L)	125.50 (111.50, 135.50)	136.00 (120.00, 146.00)	−3.506	<0.001
RBC (×10^12^/L)	4.21 (3.64, 4.53)	4.45 (3.97, 4.73)	−2.786	0.005
PLT (×10^9^/L)	216.00 (172.25, 272.75)	185.00 (135.00, 242.00)	2.871	0.004
N (×10^9^/L)	4.97 (3.79, 7.54)	4.97 (3.45, 6.94)	0.477	0.633
NEUT% (%)	71.98 ± 10.56	73.93 ± 11.03	−1.209	0.228
L (×10^9^/L)	1.14 (0.84, 1.40)	0.98 (0.65, 1.34)	1.867	0.062
LY% (%)	15.90 (10.77, 22.57)	14.50 (9.80, 22.00)	1.075	0.283
E (×10^9^/L)	0.11 (0.06, 0.23)	0.10 (0.05, 0.17)	1.179	0.238
EO% (%)	1.75 (0.90, 3.25)	1.80 (0.80, 2.50)	0.550	0.582
D-dimer (μg/mL)	0.93 (0.28, 1.30)	0.55 (0.33, 1.10)	1.396	0.163
CRP (mg/L)	14.88 (5.00, 52.75)	15.50 (5.00, 53.59)	0.294	0.768
BNP (ng/L)	484.50 (157.05, 1213.25)	588.71 (171.00, 2090.00)	−0.818	0.413
Alb (g/L)	35.90 (31.65, 38.10)	35.10 (32.00, 38.40)	0.335	0.738
Glb (g/L)	28.05 (24.40, 30.90)	26.30 (23.50, 30.20)	1.637	0.102
ALT (U/L)	14.00 (10.00, 23.75)	14.00 (9.00, 23.00)	0.325	0.745
AST (U/L)	21.00 (17.25, 27.00)	21.00 (18.00, 25.00)	0.919	0.358
BUN (μmol/L)	5.49 (4.35, 6.79)	6.17 (5.05, 7.87)	−1.992	0.046
Cr (μmol/L)	73.50 (58.25, 85.00)	74.00 (60.00, 89.00)	−0.395	0.693
UA (μmol/L)	306.16 ± 122.00	320.33 ± 121.68	−0.778	0.438
Echocardiography, electrocardiography, and pulmonary function indicators				
RV (mm)	27.00 (25.00, 29.75)	29.00 (26.00, 32.00)	−3.173	0.002
RA (mm)	28.00 (26.00, 31.00)	30.00 (27.00, 34.00)	−2.791	0.005
IVSD (mm)	10.00 (9.00, 11.75)	10.00 (9.00, 12.00)	−0.565	0.572
AO (mm)	26.00 (25.00, 30.00)	27.00 (25.00, 30.00)	−0.554	0.579
PA (mm)	19.20 (18.00, 21.00)	21.00 (18.00, 23.00)	−3.010	0.003
EF (%)	67.00 (61.25, 74.00)	65.00 (61.00, 70.00)	1.777	0.076
Right axis deviation	1 (1.1)	17 (19.1)	16.012	<0.001
Right bundle branch block	11 (12.2)	12 (13.5)	0.079	0.779
Pulmonary P wave	0 (0.0)	14 (15.7)	15.359	<0.001
FEV1%pred	44.70 (29.46, 55.90)	30.30 (24.10, 43.87)	3.286	0.001
FVC%pred	59.95 (45.85, 74.04)	51.80 (42.90, 67.58)	1.753	0.080
FEV1/FVC	53.06 (43.02, 66.54)	44.10 (36.57, 53.30)	3.312	<0.001

pH, acidity and alkalinity; PaO_2_, partial pressure of oxygen; PaCO_2_, partial pressure of carbon dioxide; SaO_2_, blood oxygen saturation; WBC, white blood cell; HB, hemoglobin; N, neutrophil; NEUT%, neutrophil percentage; L, lymphocyte; LY%, lymphocyte percentage; E, eosinophil; EO%, eosinophil percentage; D-D, D-dimer; CRP, C-reactive protein; BNP, brain natriuretic peptide; Alb, albumin; Glb, globulin; ALT, alanine aminotransferase; AST, aspartate aminotransferase; BUN, blood urea nitrogen; Cr, creatinine; UA, uric acid; RV, right ventricular diameter; RA, right atrial diameter; IVSD, interventricular septal thickness; AO, aortic diameter; PA, pulmonary artery diameter; EF, ejection fraction; FEV1%pred, forced expiratory volume in one second as a percentage of predicted value; FVC%pred, forced vital capacity as a percentage of predicted value; FEV1/FVC, ratio of forced expiratory volume in one second to forced vital capacity. Percentages were calculated using the full group denominators; tests used available observations. Two acute-exacerbation values, one drinking value, one fatigue value, one D-dimer value, and one right bundle branch block value were missing.

### Logistic regression analysis of clinical factors associated with cor pulmonale in patients with COPD

After excluding the echocardiographic and electrocardiographic variables used in the operational diagnosis, nine non-diagnostic variables were significant in univariable logistic regression and were entered simultaneously into the multivariable model (Table 2 and Supplementary Table 2). In the complete-case analysis (*n* = 177), the number of acute exacerbations in the past year (adjusted OR = 3.532, 95% CI 1.893–6.593, *P* < 0.001) and mMRC score (adjusted OR = 2.165, 95% CI 1.363–3.439, *P* = 0.001) remained positively associated with cor pulmonale. Hypertension showed an inverse association (adjusted OR = 0.333, 95% CI 0.141–0.784, *P* = 0.012), reflecting the distribution in this retrospective sample rather than a causal protective effect. PaCO_2_, oxygenation index, hemoglobin, platelet count, FEV1% predicted, and FEV1/FVC were not independently associated after mutual adjustment. Variance inflation factors ranged from 1.06 to 2.11. In the conservative sensitivity analysis excluding mMRC score, acute exacerbations remained positively associated (adjusted OR = 4.778, 95% CI 2.627–8.688, *P* < 0.001), and the inverse hypertension association persisted (adjusted OR = 0.341, 95% CI 0.149–0.778, *P* = 0.011) (Supplementary Table 3).

**TABLE 2:** Multivariable logistic regression analysis of clinical factors associated with chronic obstructive pulmonary disease (COPD) with cor pulmonale.

Indicator	B	Adjusted OR	95% CI	*P*-value
Hypertension	−1.101	0.333	(0.141, 0.784)	0.012
Number of acute exacerbations in the past year	1.262	3.532	(1.893, 6.593)	<0.001
mMRC score (grade)	0.772	2.165	(1.363, 3.439)	0.001
PaCO_2_ (mmHg)	0.029	1.029	(0.993, 1.067)	0.113
Oxygenation index (mmHg)	−0.003	0.997	(0.993, 1.002)	0.231
Hemoglobin (g/L)	0.004	1.004	(0.984, 1.025)	0.688
Platelet count (×10^9^/L)	−0.004	0.996	(0.991, 1.001)	0.100
FEV1% predicted	−0.001	0.999	(0.970, 1.028)	0.928
FEV1/FVC	−0.015	0.985	(0.944, 1.028)	0.486

### ROC analysis of independently associated clinical variables and revised clinical models

Receiver Operating Characteristic (ROC) analysis was restricted to the independently associated clinical variables and the revised multivariable models. The AUCs were 0.576 (95% CI 0.505–0.647) for absence of hypertension, 0.775 (95% CI 0.716–0.834) for acute exacerbations, and 0.782 (95% CI 0.718–0.846) for mMRC score. The revised clinical model achieved an AUC of 0.888 (95% CI 0.838–0.937). At an optimal predicted-probability threshold of 0.522, sensitivity was 79.3% and specificity was 87.8%. The model showed no evidence of poor calibration (Hosmer-Lemeshow χ^2^ = 13.113, *P* = 0.108; Brier score = 0.135). The conservative sensitivity model achieved an AUC of 0.870 (95% CI 0.816–0.923), with 79.3% sensitivity and 84.4% specificity at a threshold of 0.533 (Table 3 and Supplementary Figure 3).

**TABLE 3:** ROC analysis of independently associated clinical variables and revised clinical models.

Indicator	AUC (95% CI)	Sensitivity	Specificity	Youden index	*P*-value
Absence of hypertension	0.576 (0.505, 0.647)	0.685	0.467	0.152	0.036
Number of acute exacerbations in the past year	0.775 (0.716, 0.834)	0.471	0.911	0.382	<0.001
mMRC score	0.782 (0.718, 0.846)	0.685	0.789	0.474	<0.001
Revised clinical model	0.888 (0.838, 0.937)	0.793	0.878	0.671	<0.001
Sensitivity model excluding mMRC	0.870 (0.816, 0.923)	0.793	0.844	0.638	<0.001

### AI-assisted extraction and analysis of pulmonary vessels based on enhanced CT of the lungs

The pulmonary vessels were reconstructed through a deep convolutional neural network, and the three-dimensional images of the pulmonary arteries of each patient were obtained (Supplementary Figure 4).

The MPAD, MPAD/AOD ratio, pulmonary vessel volume indices, and mean pulmonary arterial tortuosity differed across the three groups (all *P* < 0.05 in the original vascular analysis). The normal healthy group generally had larger pulmonary vascular volume indices than the COPD groups. Consistent with Supplementary Figure 5, Vd_12_, Vd_20_, Vs_15_, and Vs_30_ mm were reported as lower in the COPD with cor pulmonale group than in the COPD-only group, whereas MPAD, MPAD/AOD, Vd_16_, and Vs_45_ mm did not differ significantly between the two COPD groups.

### Construction and evaluation of the cor pulmonale identification model

Taking the presence or absence of clinically defined cor pulmonale as the dependent variable, the small vessel volume indicators Vd_12_, Vd_20_, Vs_15_, and Vs_30_ mm reported in Table 4 and Supplementary Figure 5 were included in the multivariate Logistic regression equation to establish the cor pulmonale identification model (the relevant indicators of the model are shown in Table 5). The results indicated that the small vessel volume indicators Vd_12_, Vd_20_, Vs_15_, and Vs_30_ mm were associated with clinically defined cor pulmonale, which was consistent with clinical cor pulmonale.

**TABLE 4:** Comparison of MPAD, AOD, MPAD/AOD, pulmonary artery volume and average pulmonary artery curvature among normal group, chronic obstructive pulmonary disease (COPD) group and COPD with cor pulmonale group.

Indicator	Normal group (*n* = 31)	COPD group (*n* = 32)	COPD with cor pulmonale group (*n* = 31)	*P*-value
Vd_12__*m*_ (mm^3^)	745.61 (612.37, 912.20)	696.45 (510.41, 867.67)	452.09 (335.36, 656.71)	0.000
Vd_16__*m*_ (mm^3^)	463.24 (346.80, 652.97)	372.78 (301.00, 561.40)	316.14 (207.03, 505.17)	0.000
Vd_20__*m*_ (mm^3^)	495.19 (281.07, 513.74)	385.34 (332.98, 507.83)	279.89 (138.74, 367.68)	0.000
Vs_15__*mm*_ (mm^3^)	871.97 (432.98, 1021.04)	516.03 (332.20, 996.07)	337.19 (155.75, 514.76)	0.000
Vs_30__*mm*_ (mm^3^)	533.29 (400.80, 701.65)	432.73 (242.10, 613.07)	175.76 (92.76, 280.42)	0.000
V_*s45mm*_ (mm^3^)	582.02 (861.03, 1170.67)	506.81 (237.45, 803.25)	372.71 (214.55, 645.14)	0.000
MPAD (mm)	24.03 (22.18, 26.03)	27.65 (25.57, 29.18)	28.87 (24.69, 31.95)	0.000
AOD (mm)	30.99 (29.33, 32.07)	31.61 (29.74, 35.08)	29.99 (29.25, 31.91)	0.096
MPAD/AOD	0.77 (0.73, 0.84)	0.86 (0.80, 0.93)	0.96 (0.81, 1.06)	0.000
T (%)	0.161 (0.157, 0.171)	0.1483 (0.1422, 0.1560)	0.1429 (0.1363, 0.1549)	0.000

MPAD, main pulmonary artery diameter; AOD, aortic diameter; MPAD/AOD, ratio of main pulmonary artery diameter to aortic diameter; Vd_12_ mm, total volume of small vessels with a diameter of 12 mm; Vd_16_ mm, total volume of small vessels with a diameter of 16 mm; Vd_20_ mm, total volume of small vessels with a diameter of 20 mm; Vs_15_ mm, total volume of small vessels 15 mm away from the pleural surface; Vs_30_mm, total volume of small vessels 30 mm away from the pleural surface; Vs_45_ mm, total volume of small vessels 45 mm away from the pleural surface; T (%), average pulmonary artery curvature.

**TABLE 5:** Relevant indicators of the chronic obstructive pulmonary disease (COPD) with pulmonary arterial hypertension analysis model.

Indicator	β	SE	Wald	OR (95% confidence interval)	*P*-value
Vd_12_ mm	−0.943	0.102	2.120	0.997 (0.993, 1.001)	0.014
Vd_20_ mm	−1.335	0.156	3.422	0.996 (0.993, 1.000)	0.000
Vs_15_ mm	−2.582	0.131	2.680	0.998 (0.996, 1.000)	0.029
Vs_30_ mm	−1.547	0.014	6.993	0.994 (0.990, 0.999)	0.008
Constant	−2.094	1.494	14.530	–	0.000

**FIGURE 1:**
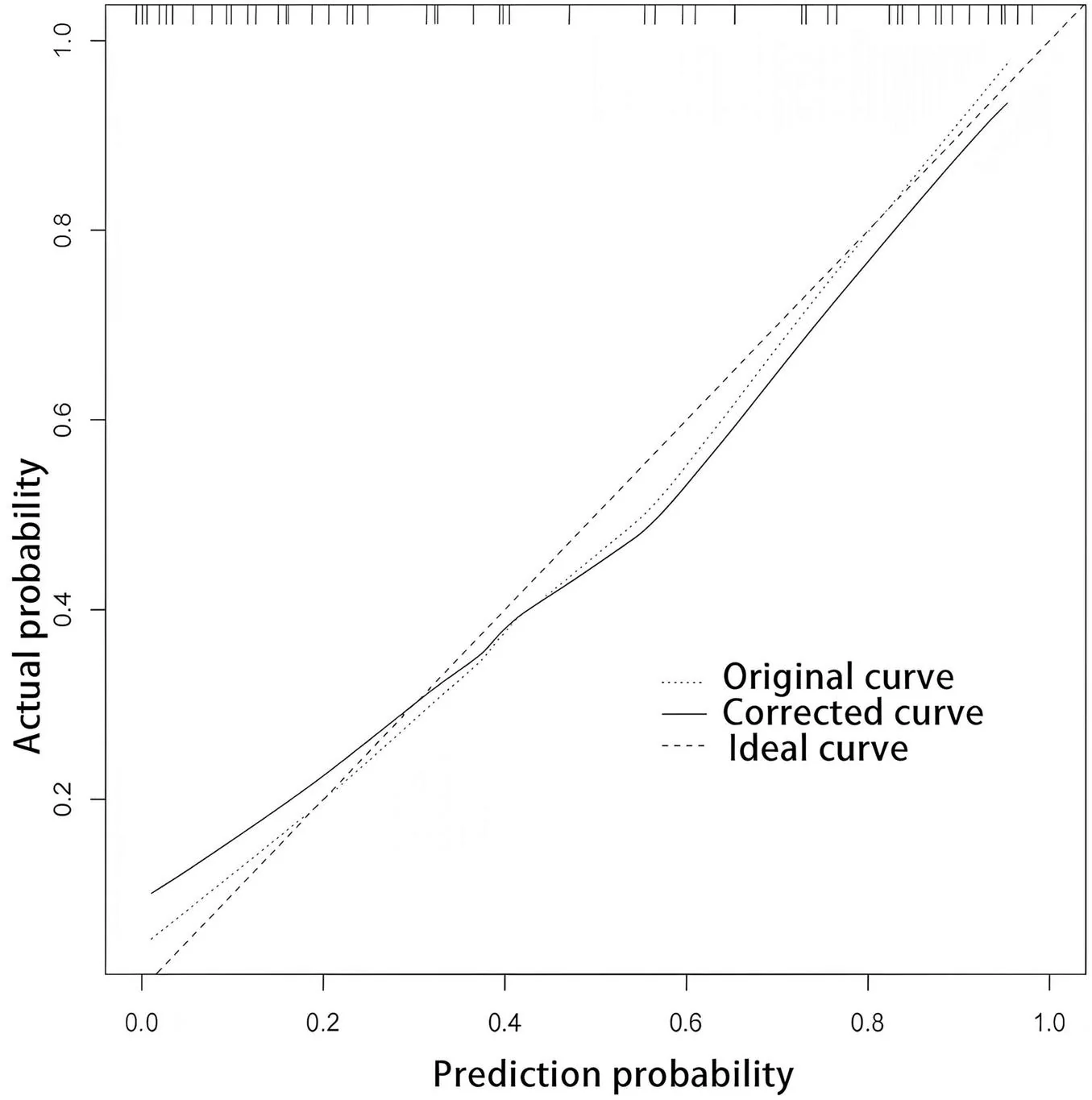
Calibration curve of the artificial intelligence (AI)-assisted vascular model for identifying clinically defined cor pulmonale in patients with chronic obstructive pulmonary disease (COPD).

The ROC curve of the model was plotted using R software, with an area under the curve of 0.895 (*P* < 0.001, 95% CI: 0.820–0.970), a specificity of 84.4%, and a sensitivity of 83.9% (Supplementary Figure 6). The Hosmer-Lemesow goodness-of-fit test was used to test the model, with χ^2^ = 4.489 and a *P*-value of 0.811, indicating no statistically significant difference and suggesting that the regression equation of the model has a strong explanatory power and a good fit between the predicted risk values and the actual observed values. To further verify the validity and stability of the model, the calibration curve was plotted using R software and the model was validated using the Bootstrap method. The results showed that the slope of the calibration curve was close to 1, indicating that the model has good predictive accuracy (Figure 1).

## Discussion

Patients with COPD and cor pulmonale had greater symptom burden, more frequent acute exacerbations, greater carbon dioxide retention, worse oxygenation, and poorer lung function than patients with COPD alone (21, 22). The revised regression analysis was designed specifically to address incorporation bias: right ventricular, right atrial, pulmonary artery, and electrocardiographic measurements that contributed to the operational diagnosis were not allowed to serve as candidate predictors. After this exclusion, acute exacerbation frequency and mMRC score remained independently and positively associated with cor pulmonale, and the combined clinical model retained good discrimination (AUC = 0.888). The sensitivity model that also removed mMRC score retained an AUC of 0.870, indicating that the clinical signal was not entirely driven by variables overlapping with the diagnostic definition. The inverse association with hypertension was consistent with the raw group distribution but is biologically unlikely to represent protection; it may reflect selection, coding, treatment, or residual confounding in this single-center retrospective sample and should be interpreted cautiously. These findings support the use of exacerbation history and symptom burden for clinical risk stratification, but they do not establish causal effects or replace hemodynamic confirmation.

Currently, non-invasive diagnostic methods for cor pulmonale in clinical practice include electrocardiogram (ECG), echocardiography, cardiac magnetic resonance imaging, and lung CT, etc. The pathological basis for diagnosing chronic cor pulmonale by ECG is right ventricular hypertrophy caused by pulmonary hypertension. ECG indicates right ventricular hypertrophy mainly based on cardiac rotation and changes in the right ventricular depolarization potential. Since the left ventricle in adults is significantly thicker than the right ventricle, if the right ventricle is only slightly hypertrophied, the depolarization potential of the left ventricle still dominates, and the ECG changes are not obvious. Only when the right ventricle is hypertrophied to a certain extent can it significantly affect the direction of the cardiac vector sum and the size of the right ventricular depolarization potential. Therefore, ECG is not sensitive for the early diagnosis of cor pulmonale (23, 24). Echocardiography can directly measure the size of the heart chambers, the thickness of the ventricular walls, and the diameter of the blood vessels, and has a high positive rate in the diagnosis of chronic cor pulmonale (25, 26). Our study also confirmed that echocardiography has higher sensitivity and specificity than ECG. Although various studies have reported high sensitivity and specificity, the inconsistent results, low precision, and potential bias risks suggest that the ability of echocardiography to assess cor pulmonale needs to be further confirmed by more high-quality studies (27). Therefore, we are still seeking a more stable non-invasive method for the early diagnosis of cor pulmonale. A recent study further demonstrated the clinical value of the CT-derived pulmonary artery-to-aorta ratio in patients with COPD (28).

Currently, the combination of deep learning technology and large pulmonary vessel volume data has made certain progress in the diagnosis and research of pulmonary arterial hypertension. Zhang et al. (29), Chu Y (30) et al. have successfully constructed models and preliminarily verified the ability of their models to diagnose PAH through large pulmonary vessels. However, the research methods were based on computed tomography pulmonary angiography (CTPA), and the study population was a broad classification of pulmonary arterial hypertension patients. The conclusions have not been specifically discussed and verified in patients with cor pulmonale, and the possibility and ability of using a model of lung small vessel volume for non-invasive diagnosis of cor pulmonale have not been explored. Multiple studies (31, 32) have found that both primary pulmonary arterial hypertension and pulmonary arterial hypertension caused by COPD have microvascular and alveolar capillary damage, with reduced lung small vessel volume, dilated main pulmonary arteries, and right ventricular dysfunction. The mechanism is blood flow redistribution caused by pulmonary vascular remodeling, and these changes often occur earlier than the onset of pulmonary arterial hypertension and cor pulmonale (33). Therefore, the reduction in lung small vessel volume has certain predictive value in the diagnosis of pulmonary arterial hypertension. In terms of imaging assessment, this study collected enhanced CT images of the lungs of patients with cor pulmonale and innovatively combined deep learning technology with enhanced CT to perform three-dimensional reconstruction of pulmonary vessels and construct a model for identifying clinically defined cor pulmonale based on small pulmonary vessel volume. The research results showed that traditional large vessel parameters (such as MPAD, MPAD/AOD) had no significant difference between the simple COPD group and the COPD with cor pulmonale group, while small vessel volume showed differences between the two groups. This result suggests that the volume of lung small arteries of different diameters (Vd_12_, Vd_20_ mm) and at different distances from the pleura (Vs_15_, Vs_30_ mm) may have potential value for identifying clinically defined cor pulmonale. This finding contrasts with the conclusion of Lambert et al.’s meta-analysis (31), which emphasized the diagnostic value of CT large vessel parameters in chronic thromboembolic pulmonary hypertension. However, this study suggests that in COPD-related pulmonary arterial hypertension, small vessel parameters may be more sensitive. This difference may be closely related to the pathological characteristics of lung small vessels in COPD patients [such as vascular spasm, compression, and remodeling (33)]. The AI model constructed in this study provides preliminary evidence for the auxiliary identification of clinically defined cor pulmonale in patients with COPD and explores new methodological approaches. Additionally, this model reduces errors caused by respiratory movement or positional deviation in traditional two-dimensional measurements by quantifying three-dimensional lung small artery volume (34, 35), with an AUC value of 0.895, and both sensitivity and specificity exceeding 83%, demonstrating high clinical application potential.

This study has several limitations. First, the sample was relatively small and derived from a single-center retrospective cohort, which increases the risks of selection bias, residual confounding, and model optimism. Second, right heart catheterization was unavailable, and the operational definition of cor pulmonale relied on retrospective echocardiographic, electrocardiographic, and clinical information. We reduced incorporation bias by excluding all objective measurements used in that definition from the predictor model and by conducting a conservative sensitivity analysis without mMRC score; nevertheless, residual incorporation bias and outcome misclassification cannot be completely excluded because clinical symptoms contributed to case ascertainment. Third, the clinical model was internally assessed in the development cohort and requires prospective external validation before clinical use. Finally, small pulmonary vessel volume measurements may be affected by vessel-wall thickness, intraluminal blood volume, image quality, and preprocessing decisions. Prospective multicenter studies incorporating right heart catheterization and prespecified image-processing procedures are needed.

## Data Availability

The original contributions presented in this study are included in this article/Supplementary material, further inquiries can be directed to the corresponding authors.

## Supplementary material

Supplementary Figure 1 **(a)** Original image; **(b)** binarized image after OTSU and adaptive thresholding; **(c)** initial vessel image after removing large connected components.
