# Supplementary material for: AI-based prediction of pulmonary hypertension in COPD patients with cor pulmonale using clinical and CT features
Source: Front Med (Lausanne). 2026 Sep 1;13:1869175. doi: 10.3389/fmed.2026.1869175 (PMC13574778; doi:10.3389/fmed.2026.1869175)
Supplement: Supplementary file 1 [file Supplementary_file_1.docx]

**Supplementary Material**

**Supplementary Figure S1 ：**obtain smaller suspected vessel regions

(a)

(b)

(c)

**Supplementary Figure S2：**shows the deep convolutional neural network for vascular

**Supplementary Figure S3. Receiver operating characteristic curves for independently associated clinical variables, the revised clinical model, and the conservative sensitivity model.**

**Supplementary Figure S4：**examples of extracted three-dimensional pulmonary vessels

**Supplementary Figure S5:**Comparison of pulmonary arteriole volume between COPD group and COPD with cor pulmonale group

**Supplementary Figure S6：** ROC Curve of the Risk Model for Pulmonary Arterial Hypertension in COPD Patients

**Supplementary Table S1：**Average values of indicators in the optimal model

| Indicator | Value |
| --- | --- |
|  | 0.8126 |
|  | 0.9981 |
|  | 0.8503 |
|  | 0.3294 |
|  | 0.4450 |
|  | 0.9980 |

**Supplementary Table S2. Univariable logistic regression analysis of candidate clinical predictors for COPD with cor pulmonale**

| **Indicator** | **B** | **OR/Exp(B)** | **95% CI** | **P value** |
| --- | --- | --- | --- | --- |
| Hypertension | -0.645 | 0.525 | (0.285, 0.965) | 0.038 |
| Number of acute exacerbations in the past year | 1.492 | 4.444 | (2.585, 7.639) | <0.001 |
| mMRC score (grade) | 1.231 | 3.425 | (2.263, 5.185) | <0.001 |
| PaCO2 (mmHg) | 0.043 | 1.044 | (1.015, 1.074) | 0.003 |
| SaO2 (%) | -0.052 | 0.949 | (0.897, 1.005) | 0.072 |
| Oxygenation index (mmHg) | -0.003 | 0.997 | (0.994, 1.000) | 0.043 |
| Hemoglobin (g/L) | 0.022 | 1.023 | (1.007, 1.038) | 0.004 |
| Red blood cell count (×10¹²/L) | -0.016 | 0.984 | (0.939, 1.032) | 0.507 |
| Platelet count (×10⁹/L) | -0.005 | 0.995 | (0.991, 0.999) | 0.007 |
| Blood urea nitrogen (μmol/L) | 0.035 | 1.036 | (0.943, 1.137) | 0.463 |
| FEV1% predicted | -0.026 | 0.974 | (0.958, 0.991) | 0.003 |
| FEV1/FVC | -0.041 | 0.960 | (0.937, 0.983) | <0.001 |

Note: Right ventricular diameter, right atrial diameter, pulmonary artery diameter, right-axis deviation, right bundle branch block, and pulmonary P wave were not evaluated as candidate predictors because they contributed to the operational definition of cor pulmonale. OR, odds ratio; CI, confidence interval.

**Supplementary Table S3. Sensitivity multivariable logistic regression excluding mMRC score**

| **Indicator** | **B** | **Adjusted OR** | **95% CI** | **P value** |
| --- | --- | --- | --- | --- |
| Hypertension | -1.077 | 0.341 | (0.149, 0.778) | 0.011 |
| Number of acute exacerbations in the past year | 1.564 | 4.778 | (2.627, 8.688) | <0.001 |
| PaCO2 (mmHg) | 0.022 | 1.022 | (0.990, 1.056) | 0.184 |
| Oxygenation index (mmHg) | -0.004 | 0.997 | (0.992, 1.001) | 0.101 |
| Hemoglobin (g/L) | 0.006 | 1.006 | (0.987, 1.026) | 0.523 |
| Platelet count (×10⁹/L) | -0.005 | 0.995 | (0.990, 1.000) | 0.052 |
| FEV1% predicted | -0.006 | 0.994 | (0.966, 1.022) | 0.668 |
| FEV1/FVC | -0.021 | 0.979 | (0.940, 1.020) | 0.312 |

Note: The sensitivity model excluded all echocardiographic and electrocardiographic variables used in the operational case definition and additionally excluded mMRC score because dyspnea contributed to clinical case ascertainment.
